# Yellow Fever Virus in Mosquitoes from Rainforest Bordering Manaus, Brazil, 2022

**DOI:** 10.3201/eid3104.240108

**Published:** 2025-04

**Authors:** Victória Bernardi, Lívia Sacchetto, Adam Hendy, Nelson F. Fé, Igor Teixeira, Beatriz de C. Marques, Kathryn A. Hanley, Maria P.G. Mourão, Marcus V.G. Lacerda, Nikos Vasilakis, Maurício L. Nogueira

**Affiliations:** São José do Rio Preto School of Medicine, São José do Rio Preto, Brazil (V. Bernardi, L. Sacchetto, I. Teixeira, B.dC. Marques, M.L. Nogueira); University of Texas Medical Branch, Galveston, Texas, USA (A. Hendy, N. Vasilakis, M.L. Nogueira); Dr Heitor Vieria Dourado Tropical Medicine Foundation, Manaus, Brazil (N.F. Fé, M.P.G. Mourão, M.V.G. Lacerda); New Mexico State University, Las Cruces, New Mexico, USA (K.A. Hanley); University of the State of Amazonas, Manaus, Brazil (M.V.G. Lacerda)

**Keywords:** Yellow fever virus, mosquitoes, rainforest, *Haemagogus*, sylvatic vectors, entomological surveillance, outbreak, Amazon basin, Brazil

## Abstract

We detected yellow fever virus in *Haemagogus* mosquitoes collected in 2022 in an Amazon rainforest bordering Manaus, Brazil. The viral genome sequence occupied a basal position within the South American I genotype 1E lineage. Our findings reinforce the Amazon Basin as a source for yellow fever virus re-emergence.

In Brazil, yellow fever virus (YFV) is transmitted in a sylvatic cycle between neotropical monkeys and canopy-dwelling *Haemagogus* and *Sabethes* spp. mosquitoes, occasionally spilling over into humans by way of bridge vectors (i.e., mosquitoes that bite both hosts) (*1*). Mandatory yellow fever (YF) vaccination combined with mosquito control initiatives have effectively eradicated the urban YF cycle; the last reported urban outbreak occurred in 1942 (*2*). Endemic to the Amazon Basin, the YFV sylvatic cycle remains the main reservoir for YFV spillover in Brazil and was the source of the 2016–2021 YF epidemic, which expanded far east and south of the basin (*2*,*3*). Because sylvatic cycles are largely impervious to human intervention and eradication (*2*), surveillance is crucial in identifying areas at risk for virus spillover.

We conducted an entomological survey from May 2021 through June 2022 at the Adolpho Ducke Forest Reserve, 100 square kilometers of primary rainforest bordering Manaus in Amazonas state, Brazil (Figure, panels A, B; Appendix Figure). We sampled mosquitoes at ground level and on 5-meter platforms with hand nets and aspirators as part of ongoing studies investigating mosquito communities at forest edges (*4*). We morphologically identified a subsample of 687 female *Haemagogus* mosquitoes as *Haemagogus janthinomys* (81%), *Hg. leucocelaenus* (12%), *Hg. baresi* (6%), or *Haemagogus* spp. (1%). We screened mosquitoes in pools of ≤10 specimens, grouped by species, site, height, and collection date (n = 378 pools; mean 1.496 [SD 1.162] specimens/pool; ) (Appendix).

**Figure:**
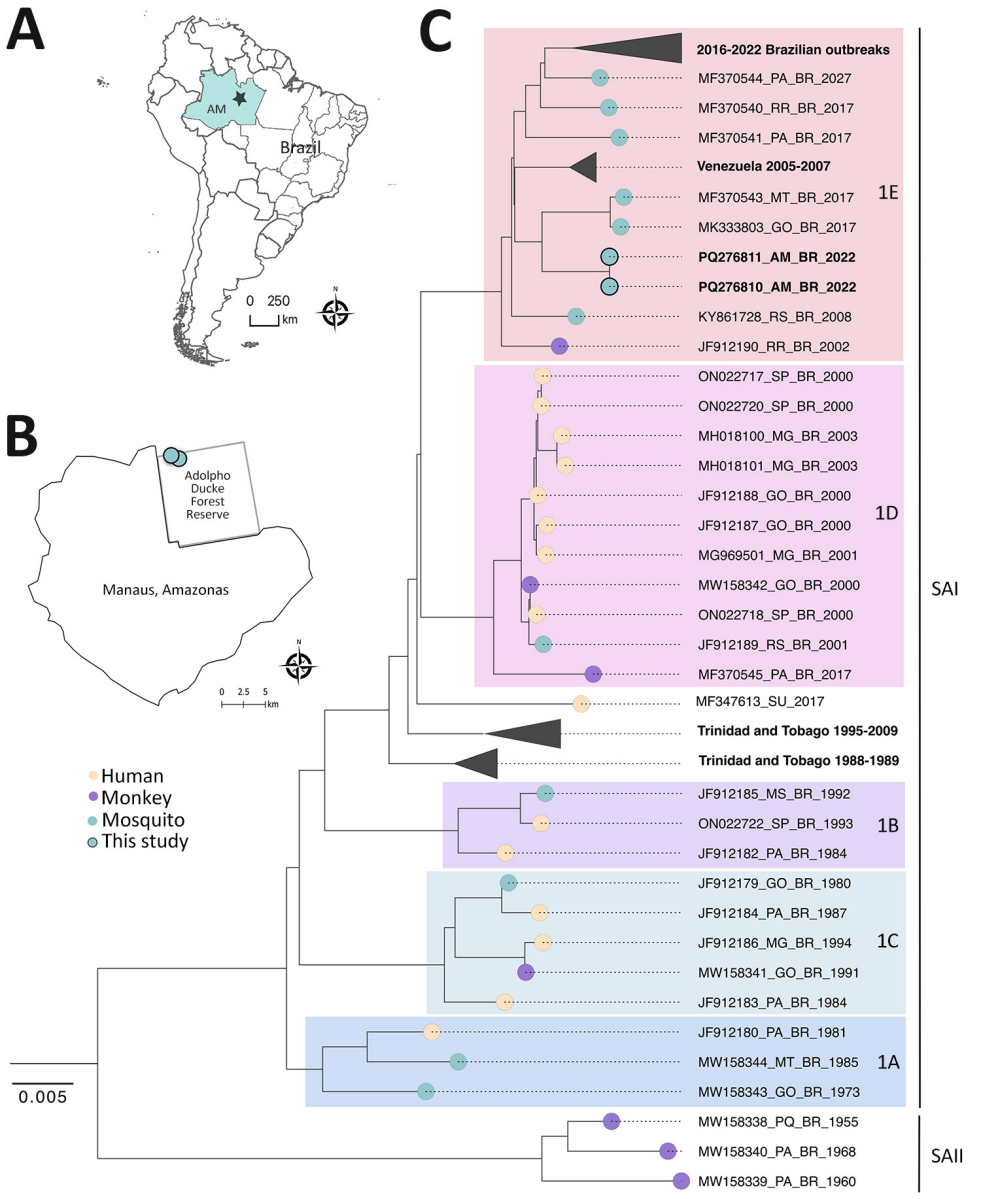
Vector surveillance at the Adolpho Ducke Forest Reserve as part of a study of yellow fever virus (YFV) in mosquitoes from rainforest bordering Manaus, Brazil, 2022. A) Location of Manaus, the capital of Amazonas state. B) Location of the Adolpho Ducke Forest Reserve bordering the city. Light green dots indicate sites where YFV-positive mosquitoes were collected in Adolpho Ducke Forest Reserve. C) Maximum-likelihood phylogenetic tree of YFV based on complete genome sequences from South America. The tree was reconstructed according to the general time reversible plus empirical base frequencies plus invariable sites plus gamma 4 nucleotide substitution model. We tested the reliability of branching patterns using ultrafast bootstrap approximation combined with a nonparametric approximate likelihood-ratio test in IQ-TREE v.2.0.3 (http://www.iqtree.org) and visualized and edited the tree in FigTree v.1.4.4 (https://github.com/rambaut/figtree). Scale bar indicates branch lengths. SAI, South American I genotype; SAII, South American II genotype; 1A–1E, SAI lineages.

We macerated samples, extracted RNA, and performed YFV-specific quantitative reverse transcription PCR (*5*). Two pools of single *Hg. janthinomys* mosquitoes collected at a treefall gap and 1 *Haemagogus* sp. mosquito collected in undisturbed forest tested positive for YFV (Figure, panel B). We morphologically identified the unclassified mosquito as *Hg. baresi*, but DNA barcoding showed its COI (cytochrome c oxidase subunit 1) sequence (GenBank accession no. PQ247126) to be 99.99% identical to a *Hg.* *janthinomys* reference sequence (accession no. NC_028025.1) as well as to the 2 morphologically identified *Hg. janthinomys* samples (accession nos. PQ247125 and PQ247127). Lacking a published COI barcode for *Hg. baresi* to contextualize this finding, we referred to this mosquito specimen as *Hg.* sp. 

All positive samples were from those collected at ground level, 500 meters interior to the forest edge, and at the end of the rainy season, in May and June 2022. We used a maximum-likelihood method to estimate infection rates of 4.37 (95% CI 1.088–11.28) per 1,000 *Haemagogus* mosquitoes (Appendix).

We performed Illumina next-generation sequencing (https://www.illumina.com) and obtained 1 complete and 1 near-complete genome from RNA extracted from the 2 *Hg. janthinomys* samples and a partial NS1 fragment (485 bp) from the *Hg.* sp. sample. We conducted genotyping using a yellow fever typing tool, which placed the sequences in the South American I (SAI) genotype. Phylogenetic analysis of the genomes sequenced placed this sequence in a basal position within the 1E lineage, closely related to sequences from the midwest region of Brazil, although not clustering in clades associated with recent YF outbreaks (2016–2022) (Figure, panel C; Appendix). We isolated YFV from this sample in C6/36 cells, which exhibited cytopathic effects (cell lysis) 6 days postinfection, confirming virus viability (Appendix).

Our study data confirm circulation of YFV near Manaus in forest-dwelling *Hg. janthinomys* mosquitoes, a sylvatic vector implicated in the most recent YF outbreaks in Brazil (*6*). *Haemagogus* mosquitoes feed primarily on monkeys but will also feed on humans (*6*), particularly at forest edges (*4*). Detection of YFV in rainforests bordering rural and periurban areas is concerning from a public health standpoint because of the comingling of humans, wildlife, and periurban and forest mosquitoes in such settings, creating the potential for arbovirus spillover (*1*). The risk of YF reurbanization remains a paramount concern given the widespread infestation of *Ae. aegypti* mosquitoes throughout South America (*2*). To date, high YFV vaccination coverage in Amazonas state (*7*) has averted outbreaks in the region, but vaccine uptake is declining (*8*). The YFV genomes we sequenced did not cluster with sequences from outbreaks in Brazil occurring in 2016–2021 and did not have the signature 9 amino acid motif associated with the latest outbreak (*9*).

Our findings emphasize the critical role of the Amazon Basin in maintaining and disseminating YFV strains to other regions of Brazil (*10*) and to neighboring countries. We sequenced the complete YFV genome from 1 *Hg. janthinomys* sample, a considerable achievement given the scarcity of genome data from the north and midwest regions of Brazil. Whole-genome sequences are crucial to understanding YFV migration dynamics in these regions (*10*). Unfortunately, we could not isolate and sequence the whole genome from the remaining 2 *Haemagogus* samples because of low viral loads, indicated by high quantitative reverse transcription PCR cycle threshold values (34.0 and 36.0). Our findings demonstrate that vector surveillance provides an early warning for arbovirus circulation, identifies high-risk areas for pathogen spillover, and guides public health efforts (vector control and vaccination) to mitigate future outbreaks.

Appendix Additional information for yellow fever virus in mosquitoes from rainforest bordering Manaus, Brazil, 2022
