# Supplementary material for: Yellow Fever Virus in Mosquitoes from Rainforest Bordering Manaus, Brazil, 2022
Source: Emerg Infect Dis. 2025 Apr;31(4):851–4. doi: 10.3201/eid3104.240108 (PMC11950274; doi:10.3201/eid3104.240108)
Supplement: Appendix — Additional information for yellow fever virus in mosquitoes from rainforest bordering Manaus, Brazil, 2022 [file 24-0108-Techapp-s1.pdf]

Article DOI: http://doi.org/10.3201/eid3104.240108

EID cannot ensure accessibility for supplementary materials supplied by authors. Readers who have difficulty accessing supplementary content should contact the authors for assistance.

Yellow Fever Virus in Mosquitoes from Rainforest Bordering Manaus, Brazil, 2022

Appendix

Materials and Methods

Mosquito collections

Mosquitoes were sampled from May 2021 to June 2022 at the Adolpho Ducke Forest Reserve, comprising 100 km^2 of primary rainforest bordering the city of Manaus in Amazonas State, Brazil (Appendix Figure 1, panels A and B). Sampling at each site occurred at ground level and on a five-meter platform to capture approaching mosquitoes using hand-nets and aspirators between 10:00 and 15:00 as part of ongoing studies investigating mosquito communities at forest edges (*1*,*2*). Mosquitoes were separated into 50 mL Falcon tubes at 30-minute intervals and tubes containing live mosquitoes were placed in a Styrofoam box in the shade to prevent desiccation until they were transferred to a −80°C freezer at the Fundação de Medicina Tropical Doutor Heitor Vieira Dourado (FMT-HVD) at the end of each day. Mosquitoes were then placed on a chill table (BioQuip, Rancho Dominguez, California, USA), morphologically identified by using a stereomicroscope and relevant taxonomic keys (*3*–*9*) and stored at −80°C until screened for the presence of viruses. Mosquito collections were approved by local environmental authorities (SISBIO license 57003–6, approved on 03.09.2020).

Homogenization of mosquito pools

Female *Haemagogus* species mosquitoes were grouped in pools of up to *10 individuals* by species, date of collection, sampling site, height, and time of collection.

Pools were macerated in 0.5 mL of phosphate-buffered saline (PBS) 1X (GIBCO, USA) supplemented with 2% penicillin-streptomycin, 1% amphotericin B, with one 5 mm stainless steel bead (Loccus, Brazil) per pool, which was shaken by using the L-BEADER 6 (Loccus,

Brazil) for three 30 second cycles at 3000 rpm. The macerated samples were centrifuged at 10,000 rpm for ten minutes, and the supernatants were transferred to a new 1.5 mL microtube.

Cell maintenance

The following cells from the American Type Culture Collection (ATCC) (Maryland, USA) were used: Vero and C6/36. The Vero cells are a continuous lineage of cells obtained by transforming green monkey kidney epithelial cells. They were grown in MEM (Eagle's Minimum Essential Medium) supplemented with 10% FBS (fetal bovine serum), containing gentamicin (50 ug/mL), penicillin (100 µL/mL) and amphotericin B (5 ug/mL), in an oven with a 5% CO^2 atmosphere at 37°C. The C6/36 cells are a lineage of epithelial cells from *Aedes albopictus* mosquito larvae. They were grown in Leibowitz-15 (L-15) medium (Cultilab, BRL), supplemented with 10% SFB, and containing 100 U/mL penicillin, 50 ug/mL streptomycin, and 2 ug/mL fungizone (Cultilab, USA). The C6/36 cells were kept in a BOD (biologic oxygen demand) incubator at 28°C.

Virus screening

A total of 300 µL of the macerated supernatants were filtered by using 0.22 µm filters attached to a 3 mL syringe, and 100 µL were inoculated per well in a 24-well plate of confluent C6/36 and Vero cells monolayers for viral isolation. The C6/36 and Vero cells were propagated and maintained in MEM or Leibowitz L-15 medium (GIBCO, USA), respectively, supplemented with 10% heat-inactivated fetal bovine serum (FBS) and 1% penicillin-streptomycin (GIBCO, USA). The adsorption period for the samples inoculated into C6/36 cells was 120 minutes, and for Vero cells, 90 minutes, rocking the plate every 15 minutes (mechanical spread of viruses over cell culture monolayers). After the adsorption period, MEM culture medium containing 1% inactivated FBS was added for Vero, and L-15 culture medium containing 2% inactivated FBS was added to C6/36 cells. The cells were incubated and observed for 7 days at 28°C (C6/36) and 37°C (Vero), respectively. After this period or when presenting a cytopathic effect, the cell culture supernatant was harvested and kept at −70°C until RNA extraction.

In parallel to in cellulo isolation, a total of 140 µL of the macerated supernatants were submitted to total RNA extraction using QIAamp Viral RNA Mini kit (Qiagen, USA), according to the manufacturer’s instructions, followed by reverse transcription-quantitative polymerase chain reaction (RT-qPCR) using GoTaq 1-Step RT-qPCR System (Promega, USA) and primers

and probes targeting the 5′ non-coding region of YFV genome at a concentration of 10µM (Appendix Table 1) (*10*). The amplification protocol consisted of an initial reverse transcription step at 45°C for 15 minutes, denaturation at 95°C for 2 minutes and 40 subsequent amplification cycles, consisting of 15 seconds at 95°C for denaturation and 1 minute at 60°C for annealing and extension. The RT-qPCR was performed on the QuantStudio 3 Real-Time PCR System (Thermo Fisher Scientific, USA), and the results were analyzed on the QuantStudio 3 v1.5.1 software (Thermo Fisher Scientific,). Results were interpreted as positive if the cycle quantification threshold (Ct) value was ≤37.

DNA Barcoding

In the positive samples, we confirmed the mosquito genus by DNA barcoding (*11*), using primers targeting the mitochondrial cytochrome c oxidase subunit I from diverse metazoan invertebrates at a concentration of 10µM (Appendix Table 2). Amplification was performed using Gotaq Hot Start Colorless Master Mix (Promega, USA) under the following thermal cycling conditions: initial denaturation at 95°C for 2 minutes, followed by 40 cycles of denaturation at 95°C for two minutes, annealing at 55°C for 1 minute, and extension at 72°C for 1 minute. A final extension was performed at 72°C for 5 minutes and the reaction was held at 4°C.

The endpoint PCR products were separated in 1.5% agarose gel in 1X TBE buffer and the gel was visualized on an ultraviolet (UV) light transilluminator. The fragments of interest were excised from the agarose and purified using the Wizard SV Gel and PCR Clean-Up System kit (Promega, USA). Purified products were subjected to sequencing reactions using the BigDye kit (Applied Biosystems, USA) and reconstituted in formamide. Sequencing was performed by capillary electrophoresis with the Sanger method *(12)* on an ABI 3130 Genetic Analyzer (Applied Biosystems).

The three mosquito sequences were analyzed using the Barcode of Life Data System (BOLD) (https://boldsystems.org). Additionally, a distance matrix analysis was performed by comparing these sequences to the complete mitochondrial genome of *Haemagogus janthinomys* (NC_028025.1).

Genome sequencing and assembly

The cDNA synthesis, YFV genome amplification, and library preparation were carried out using Illumina CovidSeq Test (Illumina Inc, USA) following the instructions provided, and adapted with a previously published primer panel for yellow fever virus South American genotype I (*13*). Sequencing was implemented on the Illumina MiSeq System (Illumina Inc, USA), using a MiSeq Reagent Kit v2 (2×150 bp cycles) (Illumina Inc, USA).

The raw sequencing data trimming was performed using Geneious Prime v. 2021.1 to filter low-quality bases, primer sequences, adapters, and reads with a minimal length of 75 bp (bp). The cleaned paired-end reads were assembled by de novo methodology using Spades v.3.15.4 (*14*). Contigs were mapped to reference (NC_002031).

Genotyping and phylogenetic analysis

Genotyping was conducted using the yellow fever typing tool available at (https://genomedetective.com/typingtool/yellowfever/). The whole genome sequence generated was combined with a total of 615 complete genomic YFV sequences retrieved from GenBank. Sequences were aligned by using MAFFT (*15*) and edited by using AliView (*16*). A maximum likelihood (ML) phylogeny tree was estimated by using IQ-TREE 2 (*17*) under the best-fit substitution model (GTR+F+I+G4) inferred by the ModelFinder application implemented in IQ-TREE2 (*18*) according to Bayesian Information Criterion (BIC). Statistical support for tree nodes was estimated using a ML bootstrap with 1,000 replicates. Visualization was performed using R software v.4.0.1.

YFV Infection Rates in Mosquito Pools

PoolTestR was used to estimate YFV infection rates in uneven sized pools of *Haemagogus* mosquitoes. Results are given as a Maximum Likelihood Estimate of the number of infected mosquitoes per 1,000 individuals with 95% confidence intervals calculated using a Likelihood Ratio Method (*19*).

Data availability

The sequences obtained in this study are available in GenBank database under accession numbers of: PQ247125-PQ247127 (COI), and PQ276810-PQ276812 (YFV).

References

1. Hendy A, Fé NF, Pedrosa I, Girão A, Figueira dos Santos TN, Mendonça CR, et al. Forest edge landscape context affects mosquito community composition and risk of pathogen emergence. 2024:2024.04.30.591911. https://doi.org/10.1101/2024.04.30.591911

2. Hendy A, Hernandez-Acosta E, Valério D, Mendonça C, Costa ER, Júnior JTA, et al. The vertical stratification of potential bridge vectors of mosquito-borne viruses in a central Amazonian forest bordering Manaus, Brazil. Sci Rep. 2020;10:18254. PubMed https://doi.org/10.1038/s41598-020-75178-3

3. Berlin O, Belkin JN. Mosquito studies (Diptera, Culicidae). XXXVI: Subgenera Aedinus, Tinolestes, and Anoedioporpa of Culex. 1980.

4. Consoli RA, Oliveira RLd. Principais mosquitos de importância sanitária no Brasil: Editora Fiocruz; 1994.

5. Forattini OP. Culicidologia médica: identificação, biologia, epidemiologia Vol. 2: Edusp; 1996.

6. Guimarães JH. Systematic database of Diptera of the Americas South of the 5. United States: family Culicidae. São Paulo, SP: Ed. Plêiade, 1997.

7. Lane J. Neotropical Culicidae, vol. 1. 1953. https://archive.org/details/biostor-59400

8. Sallum M, Forattini. Revision of the Spissipes Section of Culex (Melanoconion) (Diptera: Culicidae). 1996;12(3 Pt 2):517–600. PMID: 8887711

9. Valencia JJCAEI. Mosquito studies (Diptera, Culicidae) XXXI. A revision of the subgenus Carrollia of Culex. Contributions of the American Entomological Institute. 1973;9(4):1–134. https://www.biodiversitylibrary.org/part/382314.

10. Domingo C, Patel P, Yillah J, Weidmann M, Méndez JA, Nakouné ER, et al. Advanced yellow fever virus genome detection in point-of-care facilities and reference laboratories. J Clin Microbiol. 2012;50:4054–60. PubMed https://doi.org/10.1128/JCM.01799-12

11. Folmer O, Black M, Hoeh W, Lutz R, Vrijenhoek R. DNA primers for amplification of mitochondrial cytochrome c oxidase subunit I from diverse metazoan invertebrates. Mol Mar Biol Biotechnol. 1994;3:294–9. PubMed

12. Sanger F, Nicklen S, Coulson AR. DNA sequencing with chain-terminating inhibitors. Proc Natl Acad Sci U S A. 1977;74:5463–7. PubMed https://doi.org/10.1073/pnas.74.12.5463

13. Faria NR, Kraemer MUG, Hill SC, Goes de Jesus J, Aguiar RS, Iani FCM, et al. Genomic and epidemiological monitoring of yellow fever virus transmission potential. Science. 2018;361:894–9. PubMed https://doi.org/10.1126/science.aat7115

14. Bankevich A, Nurk S, Antipov D, Gurevich AA, Dvorkin M, Kulikov AS, et al. SPAdes: a new genome assembly algorithm and its applications to single-cell sequencing. Journal of computational biology: a journal of computational molecular cell biology. 2012;19(5):455–77.

15. Katoh K, Standley DM. MAFFT multiple sequence alignment software version 7: improvements in performance and usability. Mol Biol Evol. 2013;30:772–80. PubMed https://doi.org/10.1093/molbev/mst010

16. Larsson A. AliView: a fast and lightweight alignment viewer and editor for large datasets. Bioinformatics. 2014;30:3276–8. PubMed https://doi.org/10.1093/bioinformatics/btu531

17. Nguyen LT, Schmidt HA, von Haeseler A, Minh BQ. IQ-TREE: a fast and effective stochastic algorithm for estimating maximum-likelihood phylogenies. Mol Biol Evol. 2015;32:268–74. PubMed https://doi.org/10.1093/molbev/msu300

18. Minh BQ, Schmidt HA, Chernomor O, Schrempf D, Woodhams MD, von Haeseler A, et al. IQ-TREE 2: New Models and Efficient Methods for Phylogenetic Inference in the Genomic Era. Mol Biol Evol. 2020;37:1530–4. PubMed https://doi.org/10.1093/molbev/msaa015

19. McLure A, O'Neill B, Mayfield H, Lau C, McPherson B. PoolTestR: An R package for estimating prevalence and regression modelling for molecular xenomonitoring and other applications with pooled samples. Environ Model Softw. 2021;145:105158. https://doi.org/10.1016/j.envsoft.2021.105158

Appendix Table 1. Primers and probes for YFV RT-qPCR.

Primer/probe	Sequence (5′-3′)	Position[a]
YFallF	5′-GCTAATTGAGGTGYATTGGTCTGC-3′	15–38
YFallR	5′-CTGCTAATCGCTCAAMGAACG-3′	83–103
YFallP	5′-HEX-ATCGAGTTGCTAGGCAATAAACAC-TMR-3′	41–64

[a]Positions are indicated relative to GenBank sequence AY640589.1.

Appendix Table 2. Primers for mitochondrial cytochrome c oxidase subunit I from diverse metazoan invertebrates.

Primer	Sense	Sequence (5′-3′)	Size (bp)
LCO1490	Forward	GGTCAACAAATCATAAAGATATTGG	710
HC02198	Reverse	TAAACTTCAGGGTGACCAAAAAATCA	710

Appendix Table 3. Yellow fever virus genome sequences used in the phylogenetic analysis.

GenBank Acession	Country	Year	Source	GenBank Acession	Country	Year	Source
**OP508690.1**	Brazil	2021	Alouatta sp	**MH484430.1**	Brazil	2017	Callithrix sp
**OP508660.1**	Brazil	2021	Alouatta sp	**MF170971.1**	Brazil	2017	New World monkey
**OP508668.1**	Brazil	2021	Alouatta sp	**MH018115.1**	Brazil	2017	Non-human primate
**MZ712144.1**	Brazil	2021	Alouatta guariba clamitans	**MK333804.1**	Brazil	2015	Sapajus libidinosus
**OP508691.1**	Brazil	2021	Alouatta sp	**MK728873.1**	Brazil	2017	Homo sapiens
**MZ712134.1**	Brazil	2021	Alouatta guariba clamitans	**ON022275.1**	Brazil	2018	Homo sapiens
**MZ712133.1**	Brazil	2021	Alouatta guariba clamitans	**ON022274.1**	Brazil	2018	Homo sapiens
**MZ712143.1**	Brazil	2021	Alouatta guariba clamitans	**ON022238.1**	Brazil	2017	Non-human primate
**MZ712140.1**	Brazil	2021	Alouatta guariba clamitans	**ON022239.1**	Brazil	2018	Homo sapiens
**MZ712132.1**	Brazil	2021	Alouatta guariba clamitans	**ON022276.1**	Brazil	2018	Homo sapiens
**MZ712127.1**	Brazil	2021	Alouatta guariba clamitans	**MK333809.1**	Brazil	2018	Haemagogus janthinomys
**MZ712146.1**	Brazil	2021	Alouatta guariba clamitans	**MK333807.1**	Brazil	2018	Haemagogus leucocelaenus
**OP508655.1**	Brazil	2021	Alouatta sp	**ON502416.1**	Brazil	2018	Homo sapiens
**OP508659.1**	Brazil	2021	Alouatta sp	**MK333806.1**	Brazil	2018	Haemagogus janthinomys
**OP508672.1**	Brazil	2021	Alouatta sp	**ON022242.1**	Brazil	2018	Homo sapiens
**OP508657.1**	Brazil	2021	NA	**MN506290.1**	Brazil	2018	Haemagogus janthinomys
**MZ712130.1**	Brazil	2021	Alouatta guariba clamitans	**ON022271.1**	Brazil	2018	Homo sapiens
**MZ712128.1**	Brazil	2021	Alouatta guariba clamitans	**ON022270.1**	Brazil	2018	Homo sapiens
**MZ712129.1**	Brazil	2021	Alouatta guariba clamitans	**MN643077.1**	Brazil	2018	Homo sapiens
**MZ712138.1**	Brazil	2021	Alouatta guariba clamitans	**ON022508.1**	Brazil	2018	Homo sapiens
**MZ712131.1**	Brazil	2021	Alouatta guariba clamitans	**ON022498.1**	Brazil	2018	Homo sapiens
**OP508709.1**	Brazil	2021	Alouatta guariba clamitans	**ON022729.1**	Brazil	2018	Homo sapiens
**OP508697.1**	Brazil	2021	Alouatta sp	**ON022483.1**	Brazil	2018	Homo sapiens
**OP508682.1**	Brazil	2019	Alouatta sp	**ON022476.1**	Brazil	2018	Homo sapiens
**OP508699.1**	Brazil	2021	Alouatta sp	**ON022492.1**	Brazil	2018	Homo sapiens
**OP508693.1**	Brazil	2021	Alouatta sp	**ON022506.1**	Brazil	2018	Homo sapiens
**OP508683.1**	Brazil	2021	Alouatta sp	**ON022500.1**	Brazil	2018	Homo sapiens
**OP508689.1**	Brazil	2021	Alouatta sp	**ON022448.1**	Brazil	2018	Callithrix
**OP508679.1**	Brazil	2021	Alouatta sp	**ON022420.1**	Brazil	2017	Alouatta caraya
**MZ712147.1**	Brazil	2021	Alouatta sp	**ON022424.1**	Brazil	2017	Alouatta guariba clamitans
**MZ712148.1**	Brazil	2021	Alouatta guariba clamitans	**MN643080.1**	Brazil	2018	Homo sapiens
**MZ712137.1**	Brazil	2021	Alouatta guariba clamitans	**MN506265.1**	Brazil	2017	Haemagogus janthinomys
**MZ712149.1**	Brazil	2021	Alouatta guariba clamitans	**ON022240.1**	Brazil	2018	Alouatta sp
**OP508685.1**	Brazil	2021	Alouatta sp	**MN643078.1**	Brazil	2018	Homo sapiens
**OP508704.1**	Brazil	2021	Alouatta sp	**MN604284.1**	Brazil	2017	Homo sapiens
**OP508686.1**	Brazil	2021	Alouatta sp	**ON022471.1**	Brazil	2018	Homo sapiens
**OP508680.1**	Brazil	2021	Alouatta sp	**ON022450.1**	Brazil	2018	Callithrix
**OP508702.1**	Brazil	2021	Alouatta sp	**MK882619.1**	Brazil	2018	Homo sapiens
**OP508695.1**	Brazil	2021	Alouatta sp	**ON022280.1**	Brazil	2017	Alouatta sp
**OP508677.1**	Brazil	2021	Alouatta sp	**MK882604.1**	Brazil	2017	Homo sapiens
**OP508674.1**	Brazil	2021	Alouatta sp	**MN643079.1**	Brazil	2018	Homo sapiens

GenBank Acession	Country	Year	Source	GenBank Acession	Country	Year	Source
**OP508700.1**	Brazil	2020	Alouatta sp	**ON022281.1**	Brazil	2017	Alouatta sp
**OP508692.1**	Brazil	2021	Alouatta sp	**MF423377.2**	Brazil	2017	Alouatta guariba clamitans
**OP508678.1**	Brazil	2021	Alouatta sp	**MF538785.2**	Brazil	2017	marmoset
**OP508684.1**	Brazil	2021	Alouatta sp	**MF370548.1**	Brazil	2017	Alouatta sp
**OP508705.1**	Brazil	2021	Alouatta sp	**MF170975.1**	Brazil	2017	Homo sapiens
**OP508713.1**	Brazil	2021	Alouatta sp	**MK882610.1**	Brazil	2017	NA
**OP508646.1**	Brazil	2020	Alouatta sp	**MF423373.2**	Brazil	2017	Haemagogus leucocelaenus
**OP508698.1**	Brazil	2021	Alouatta sp	**MF423378.2**	Brazil	2017	Alouatta guariba clamitans
**MZ712139.1**	Brazil	2021	Alouatta guariba clamitans	**MN506291.1**	Brazil	2017	Haemagogus janthinomys
**OP508648.1**	Brazil	2020	Alouatta sp	**MN506285.1**	Brazil	2018	Haemagogus janthinomys
**OP508638.1**	Brazil	2020	Alouatta sp	**MN506284.1**	Brazil	2018	Haemagogus janthinomys
**OP508606.1**	Brazil	2020	Alouatta sp	**MN506289.1**	Brazil	2018	Haemagogus janthinomys
**OP508619.1**	Brazil	2020	Alouatta sp	**MN506287.1**	Brazil	2018	Haemagogus janthinomys
**OP508645.1**	Brazil	2020	Alouatta sp	**MN506286.1**	Brazil	2018	Haemagogus janthinomys
**OP508636.1**	Brazil	2020	Alouatta sp	**MK882618.1**	Brazil	2018	Homo sapiens
**OP508701.1**	Brazil	2021	Alouatta sp	**MN506288.1**	Brazil	2018	Haemagogus janthinomys
**OP508707.1**	Brazil	2021	Alouatta sp	**ON022439.1**	Brazil	2017	Alouatta sp
**OP508676.1**	Brazil	2019	Alouatta sp	**ON022382.1**	Brazil	2017	Alouatta caraya
**OP508710.1**	Brazil	2021	Alouatta sp	**ON022264.1**	Brazil	2017	Callithrix penicillata
**OP508670.1**	Brazil	2021	Alouatta sp	**ON022513.1**	Brazil	2018	Homo sapiens
**OP508669.1**	Brazil	2021	Alouatta sp	**ON022494.1**	Brazil	2018	Homo sapiens
**OP508664.1**	Brazil	2021	Alouatta sp	**ON022479.1**	Brazil	2018	Homo sapiens
**OP508603.1**	Brazil	2019	Alouatta sp	**ON022491.1**	Brazil	2018	Homo sapiens
**OP508599.1**	Brazil	2019	Alouatta sp	**ON022510.1**	Brazil	2018	Homo sapiens
**OP508628.1**	Brazil	2020	Alouatta sp	**ON022499.1**	Brazil	2018	Homo sapiens
**OP508662.1**	Brazil	2021	Alouatta sp	**ON022503.1**	Brazil	2018	Homo sapiens
**OP508623.1**	Brazil	2020	Alouatta sp	**ON022473.1**	Brazil	2018	Homo sapiens
**OP508637.1**	Brazil	2020	Alouatta guariba clamitans	**ON022502.1**	Brazil	2018	Homo sapiens
**OP508635.1**	Brazil	2020	Alouatta sp	**ON022522.1**	Brazil	2018	Homo sapiens
**OP508632.1**	Brazil	2020	Alouatta sp	**ON022534.1**	Brazil	2018	Homo sapiens
**OP508612.1**	Brazil	2020	Alouatta sp	**ON022727.1**	Brazil	2018	Homo sapiens
**OP508620.1**	Brazil	2020	Alouatta sp	**ON022489.1**	Brazil	2018	Homo sapiens
**OP508626.1**	Brazil	2020	Alouatta sp	**ON022501.1**	Brazil	2018	Homo sapiens
**OP508610.1**	Brazil	2020	Alouatta sp	**ON022493.1**	Brazil	2018	Homo sapiens
**OP508663.1**	Brazil	2021	Alouatta sp	**ON022517.1**	Brazil	2018	Callithrix
**OP508642.1**	Brazil	2020	Alouatta sp	**ON022465.1**	Brazil	2018	Callithrix
**OP508618.1**	Brazil	2020	Alouatta sp	**ON022466.1**	Brazil	2017	Alouatta sp
**OP508614.1**	Brazil	2019	Alouatta sp	**ON022519.1**	Brazil	2017	Callithrix
**OP508607.1**	Brazil	2020	Alouatta sp	**ON022460.1**	Brazil	2017	Alouatta sp
**OP508696.1**	Brazil	2020	Alouatta sp	**MK333808.1**	Brazil	2018	Haemagogus janthinomys
**MZ712136.1**	Brazil	2021	Alouatta guariba clamitans	**ON022516.1**	Brazil	2018	Homo sapiens
**MZ712135.1**	Brazil	2021	Alouatta guariba clamitans	**ON022273.1**	Brazil	2018	Homo sapiens

GenBank Acession	Country	Year	Source	GenBank Acession	Country	Year	Source
**OP508665.1**	Brazil	2021	Alouatta sp	**ON022278.1**	Brazil	2018	Homo sapiens
**MZ712142.1**	Brazil	2021	Alouatta guariba clamitans	**ON022397.1**	Brazil	2017	Alouatta guariba clamitans
**OP508643.1**	Brazil	2020	Alouatta sp	**ON022438.1**	Brazil	2018	Alouatta sp
**MZ712141.1**	Brazil	2021	Alouatta guariba clamitans	**ON022474.1**	Brazil	2018	Homo sapiens
**OP508681.1**	Brazil	2020	Alouatta sp	**ON022537.1**	Brazil	2017	Homo sapiens
**OP508600.1**	Brazil	2019	Alouatta sp	**ON022467.1**	Brazil	2017	Callithrix
**OP508617.1**	Brazil	2020	Alouatta sp	**ON022430.1**	Brazil	2017	Alouatta caraya
**OP508631.1**	Brazil	2020	Non-human primate (NHP)	**ON022463.1**	Brazil	2017	Alouatta sp
**OP508621.1**	Brazil	2020	Alouatta sp	**ON022383.1**	Brazil	2017	Cebidae
**OP508687.1**	Brazil	2021	Alouatta sp	**MH018077.1**	Brazil	2017	Homo sapiens
**OP508587.1**	Brazil	2019	Homo sapiens	**MH018070.1**	Brazil	2017	Homo sapiens
**OP508630.1**	Brazil	2020	Alouatta sp	**MH018085.1**	Brazil	2017	Homo sapiens
**OP508608.1**	Brazil	2020	Alouatta sp	**MH018076.1**	Brazil	2017	Homo sapiens
**OP508602.1**	Brazil	2019	Alouatta sp	**MH018083.1**	Brazil	2017	Alouatta sp
**ON022559.1**	Brazil	2019	Homo sapiens	**ON022509.1**	Brazil	2018	Homo sapiens
**MZ604869.1**	Brazil	2019	Homo sapiens	**ON022532.1**	Brazil	2017	Homo sapiens
**MZ604841.1**	Brazil	2019	Homo sapiens	**MH018092.1**	Brazil	2017	Homo sapiens
**OP508716.1**	Brazil	2021	Alouatta sp	**ON022387.1**	Brazil	2017	Alouatta caraya
**OP508666.1**	Brazil	2021	Alouatta sp	**ON022449.1**	Brazil	2018	Callithrix
**OP508658.1**	Brazil	2021	Alouatta sp	**ON022475.1**	Brazil	2018	Homo sapiens
**OP508653.1**	Brazil	2021	Alouatta sp	**ON022445.1**	Brazil	2018	Callithrix
**OP508652.1**	Brazil	2021	Alouatta sp	**ON022507.1**	Brazil	2018	Homo sapiens
**OP508650.1**	Brazil	2021	Alouatta sp	**ON022485.1**	Brazil	2018	Homo sapiens
**OP508654.1**	Brazil	2021	Alouatta sp	**ON022461.1**	Brazil	2017	Non-human primate
**OP508711.1**	Brazil	2021	Alouatta sp	**ON022735.1**	Brazil	2017	Callithrix
**MZ712145.1**	Brazil	2021	Alouatta guariba clamitans	**ON022734.1**	Brazil	2017	Callithrix
**OP508647.1**	Brazil	2020	Alouatta sp	**ON022512.1**	Brazil	2018	Homo sapiens
**OP508714.1**	Brazil	2021	Alouatta sp	**ON022543.1**	Brazil	2018	Homo sapiens
**OP508712.1**	Brazil	2021	Alouatta sp	**ON022486.1**	Brazil	2018	Homo sapiens
**OP508649.1**	Brazil	2020	Alouatta sp	**ON022452.1**	Brazil	2018	Alouatta sp
**OP508651.1**	Brazil	2020	Alouatta sp	**ON022539.1**	Brazil	2017	Homo sapiens
**OP508656.1**	Brazil	2021	Alouatta sp	**ON022660.1**	Brazil	2018	Non-human primate
**OP508708.1**	Brazil	2021	Alouatta sp	**ON022442.1**	Brazil	2018	Callithrix
**OP508667.1**	Brazil	2021	Alouatta sp	**ON022437.1**	Brazil	2018	Callithrix
**OP508627.1**	Brazil	2020	Alouatta sp	**ON022454.1**	Brazil	2018	Alouatta sp
**OP508624.1**	Brazil	2020	Alouatta sp	**ON022481.1**	Brazil	2018	Homo sapiens
**OP508625.1**	Brazil	2020	Alouatta sp	**ON022526.1**	Brazil	2018	Homo sapiens
**OP508609.1**	Brazil	2020	Alouatta sp	**ON022468.1**	Brazil	2017	Non-human primate
**OP508640.1**	Brazil	2020	Alouatta sp	**ON022724.1**	Brazil	2018	Homo sapiens
**OP508706.1**	Brazil	2020	Allouatta clamitans	**ON022533.1**	Brazil	2018	Homo sapiens
**OP508615.1**	Brazil	2020	Alouatta guariba clamitans	**ON022520.1**	Brazil	2017	Homo sapiens
**OP508703.1**	Brazil	2021	Alouatta sp	**ON022544.1**	Brazil	2018	Homo sapiens

GenBank Acession	Country	Year	Source	GenBank Acession	Country	Year	Source
**OP508616.1**	Brazil	2020	Alouatta guariba clamitans	**ON022733.1**	Brazil	2018	Callithrix
**MZ604839.1**	Brazil	2019	Homo sapiens	**ON022530.1**	Brazil	2018	Non-human primate
**MZ604846.1**	Brazil	2019	Homo sapiens	**MH018086.1**	Brazil	2017	Homo sapiens
**ON022549.1**	Brazil	2019	Homo sapiens	**MH018075.1**	Brazil	2017	Homo sapiens
**MZ604853.1**	Brazil	2019	Homo sapiens	**MH018097.1**	Brazil	2017	Callicebus sp
**OP508591.1**	Brazil	2019	Homo sapiens	**MH018093.1**	Brazil	2017	Homo sapiens
**OP508594.1**	Brazil	2019	Alouatta sp	**MH018074.1**	Brazil	2017	Homo sapiens
**OP508598.1**	Brazil	2019	Alouatta sp	**MH018089.1**	Brazil	2017	Homo sapiens
**OP508595.1**	Brazil	2019	Alouatta sp	**MH018084.1**	Brazil	2017	Callithrix sp.
**OP508596.1**	Brazil	2019	Alouatta sp	**MH018073.1**	Brazil	2017	Homo sapiens
**OP508675.1**	Brazil	2019	Hg janthinomys/capricornii	**MH018072.1**	Brazil	2017	Homo sapiens
**OP508593.1**	Brazil	2019	Alouatta sp	**MH018087.1**	Brazil	2017	Homo sapiens
**OP508688.1**	Brazil	2019	Alouatta sp	**MH018088.1**	Brazil	2017	Homo sapiens
**MZ604871.1**	Brazil	2019	Homo sapiens	**ON022723.1**	Brazil	2017	Non-human primate
**MZ604857.1**	Brazil	2019	Homo sapiens	**ON022732.1**	Brazil	2017	Non-human primate
**ON022548.1**	Brazil	2019	Homo sapiens	**MH018098.1**	Brazil	2017	Cebidae sp
**ON022369.1**	Brazil	2019	Non-human primate	**MH018081.1**	Brazil	2017	Cebidae sp
**ON022658.1**	Brazil	2018	Alouatta sp	**ON022731.1**	Brazil	2017	Homo sapiens
**ON022562.1**	Brazil	2019	Homo sapiens	**ON022726.1**	Brazil	2018	Homo sapiens
**OP508597.1**	Brazil	2019	Alouatta sp	**ON022529.1**	Brazil	2017	Homo sapiens
**OP508592.1**	Brazil	2019	Alouatta sp	**ON022469.1**	Brazil	2017	Callithrix
**ON022561.1**	Brazil	2019	Homo sapiens	**ON022730.1**	Brazil	2017	Homo sapiens
**ON022558.1**	Brazil	2019	Homo sapiens	**ON022528.1**	Brazil	2017	Homo sapiens
**ON022555.1**	Brazil	2019	Homo sapiens	**ON022514.1**	Brazil	2018	Homo sapiens
**ON022546.1**	Brazil	2019	Homo sapiens	**ON022511.1**	Brazil	2018	Homo sapiens
**ON022552.1**	Brazil	2019	Homo sapiens	**ON022427.1**	Brazil	2017	Alouatta guariba clamitans
**MZ604847.1**	Brazil	2019	Homo sapiens	**ON022462.1**	Brazil	2018	Callithrix
**MZ604863.1**	Brazil	2019	Homo sapiens	**ON022413.1**	Brazil	2017	Alouatta guariba clamitans
**MZ604875.1**	Brazil	2019	Homo sapiens	**ON022406.1**	Brazil	2017	Alouatta guariba clamitans
**MZ604838.1**	Brazil	2019	Homo sapiens	**ON022432.1**	Brazil	2017	Alouatta guariba clamitans
**MZ604843.1**	Brazil	2019	Homo sapiens	**ON022392.1**	Brazil	2018	Callithrix
**MZ604844.1**	Brazil	2019	Homo sapiens	**ON022497.1**	Brazil	2018	Homo sapiens
**MW308135.1**	Brazil	2019	Homo sapiens	**ON022443.1**	Brazil	2017	Alouatta
**ON022319.1**	Brazil	2019	Homo sapiens	**ON022398.1**	Brazil	2017	Alouatta guariba clamitans
**MZ604864.1**	Brazil	2019	Homo sapiens	**ON022523.1**	Brazil	2017	Homo sapiens
**MZ604854.1**	Brazil	2019	Homo sapiens	**ON022524.1**	Brazil	2017	Homo sapiens
**ON022306.1**	Brazil	2018	Alouatta sp	**ON022409.1**	Brazil	2017	Alouatta caraya
**MH030059.1**	Brazil	2017	Alouatta sp	**ON022416.1**	Brazil	2017	Alouatta caraya
**ON022308.1**	Brazil	2018	Alouatta sp	**ON022538.1**	Brazil	2017	Homo sapiens
**ON022583.1**	Brazil	2018	Alouatta sp	**ON022527.1**	Brazil	2017	Homo sapiens
**ON022595.1**	Brazil	2018	Alouatta sp	**ON022535.1**	Brazil	2017	Homo sapiens
**ON022307.1**	Brazil	2018	Homo sapiens	**ON022525.1**	Brazil	2017	Homo sapiens

GenBank Acession	Country	Year	Source	GenBank Acession	Country	Year	Source
**ON022309.1**	Brazil	2018	Alouatta sp	**ON022536.1**	Brazil	2017	Homo sapiens
**MW308134.1**	Brazil	2018	Homo sapiens	**ON022399.1**	Brazil	2017	Cebidae
**MZ604850.1**	Brazil	2019	Homo sapiens	**MH484432.1**	Brazil	2017	Alouatta sp
**MZ604858.1**	Brazil	2019	Homo sapiens	**ON022531.1**	Brazil	2017	Homo sapiens
**MZ604860.1**	Brazil	2019	Homo sapiens	**MH018090.1**	Brazil	2017	Homo sapiens
**MZ604867.1**	Brazil	2019	Homo sapiens	**ON022456.1**	Brazil	2017	Non-human primate
**MZ604849.1**	Brazil	2019	Homo sapiens	**MK882601.1**	Brazil	2017	Homo sapiens
**MZ604851.1**	Brazil	2019	Homo sapiens	**ON022455.1**	Brazil	2017	Alouatta sp
**MZ604852.1**	Brazil	2019	Homo sapiens	**ON022258.1**	Brazil	2017	Cebidae
**MZ604874.1**	Brazil	2019	Homo sapiens	**ON022265.1**	Brazil	2018	Callithrix penicillata
**MZ604848.1**	Brazil	2019	Homo sapiens	**ON022268.1**	Brazil	2018	Homo sapiens
**MZ604855.1**	Brazil	2019	Homo sapiens	**ON022423.1**	Brazil	2017	Callithrix penicillata
**ON022295.1**	Brazil	2018	Haemagogus leucocelaenus	**ON022262.1**	Brazil	2018	Alouatta sp
**MK583151.1**	Brazil	2018	Homo sapiens	**ON502413.1**	Brazil	2018	Homo sapiens
**MK333798.1**	Brazil	2018	Homo sapiens	**ON502399.1**	Brazil	2018	Homo sapiens
**MH193173.1**	Brazil	2018	Alouatta sp	**ON022257.1**	Brazil	2018	Homo sapiens
**ON022567.1**	Brazil	2017	Alouatta sp	**ON022263.1**	Brazil	2018	Homo sapiens
**MH030063.1**	Brazil	2017	Alouatta sp	**ON502391.1**	Brazil	2018	Homo sapiens
**MH030065.1**	Brazil	2017	Alouatta sp	**ON502438.1**	Brazil	2018	Homo sapiens
**ON022352.1**	Brazil	2018	Alouatta sp	**ON502389.1**	Brazil	2018	Homo sapiens
**MH030061.1**	Brazil	2017	Alouatta sp	**ON022728.1**	Brazil	2018	Homo sapiens
**MH030060.1**	Brazil	2017	Alouatta sp	**ON022495.1**	Brazil	2018	Homo sapiens
**MH030062.1**	Brazil	2017	Alouatta sp	**ON022725.1**	Brazil	2017	Homo sapiens
**ON022337.1**	Brazil	2018	Alouatta sp	**ON022428.1**	Brazil	2017	Alouatta caraya
**MH030064.1**	Brazil	2017	Alouatta sp	**ON022425.1**	Brazil	2017	Callithrix penicillata
**ON022330.1**	Brazil	2018	Alouatta sp	**ON022385.1**	Brazil	2017	Callithrix penicillata
**MH030050.1**	Brazil	2017	Callicebus sp	**ON022405.1**	Brazil	2017	Callithrix penicillata
**MK583147.1**	Brazil	2018	Homo sapiens	**ON022411.1**	Brazil	2017	Callithrix penicillata
**MK583155.1**	Brazil	2018	Homo sapiens	**ON022431.1**	Brazil	2017	Cebidae
**MZ604845.1**	Brazil	2018	Homo sapiens	**ON022373.1**	Brazil	2017	Alouatta caraya
**ON022743.1**	Brazil	2018	Homo sapiens	**MK249066.1**	Brazil	2018	Non-human primate
**ON022745.1**	Brazil	2018	Homo sapiens	**ON502398.1**	Brazil	2018	Homo sapiens
**ON022645.1**	Brazil	2017	Cebus	**ON502409.1**	Brazil	2018	Homo sapiens
**ON022644.1**	Brazil	2017	Callithrix	**ON022266.1**	Brazil	2017	Cebidae
**ON022628.1**	Brazil	2017	Alouatta sp	**ON502414.1**	Brazil	2018	Homo sapiens
**ON022631.1**	Brazil	2017	Alouatta sp	**ON502392.1**	Brazil	2018	Homo sapiens
**ON022636.1**	Brazil	2017	Callicebus	**ON502419.1**	Brazil	2018	Homo sapiens
**ON022634.1**	Brazil	2017	Alouatta sp	**ON022260.1**	Brazil	2018	Homo sapiens
**ON022642.1**	Brazil	2017	Alouatta sp	**ON502396.1**	Brazil	2018	Homo sapiens
**MH030051.1**	Brazil	2017	Alouatta sp	**ON022259.1**	Brazil	2018	Callithrix penicillata
**ON022356.1**	Brazil	2017	Alouatta sp	**ON502393.1**	Brazil	2018	Homo sapiens
**ON022353.1**	Brazil	2017	Alouatta sp	**ON502410.1**	Brazil	2018	Homo sapiens

GenBank Acession	Country	Year	Source	GenBank Acession	Country	Year	Source
**ON022360.1**	Brazil	2018	Alouatta sp	**ON502394.1**	Brazil	2018	Homo sapiens
**ON022359.1**	Brazil	2017	Alouatta sp	**ON502442.1**	Brazil	2018	Homo sapiens
**ON022354.1**	Brazil	2017	Alouatta sp	**ON502412.1**	Brazil	2018	Homo sapiens
**ON022355.1**	Brazil	2017	Alouatta sp	**ON022422.1**	Brazil	2017	Cebidae
**MZ604872.1**	Brazil	2018	Homo sapiens	**ON022417.1**	Brazil	2017	Alouatta guariba clamitans
**MZ604859.1**	Brazil	2018	Homo sapiens	**MH484426.1**	Brazil	2017	Non-human primate
**MK583166.1**	Brazil	2018	Homo sapiens	**MH484434.1**	Brazil	2017	Homo sapiens
**MK333799.1**	Brazil	2018	Homo sapiens	**MH484427.1**	Brazil	2017	Callithrix sp
**ON022342.1**	Brazil	2018	Callithrix	**ON022384.1**	Brazil	2017	Callithrix geoffroyi
**MK583163.1**	Brazil	2018	Homo sapiens	**ON022415.1**	Brazil	2017	Callithrix geoffroyi
**MH030049.1**	Brazil	2017	Callithrix	**ON022379.1**	Brazil	2017	Non-human primate
**ON022714.1**	Brazil	2017	Non-human primate	**ON022388.1**	Brazil	2017	Callithrix geoffroyi
**ON022598.1**	Brazil	2018	Non-human primate	**ON022375.1**	Brazil	2017	Cebidae
**ON022709.1**	Brazil	2017	Non-human primate	**MF538784.2**	Brazil	2017	Homo sapiens
**ON022659.1**	Brazil	2018	Alouatta sp	**MF170977.1**	Brazil	2017	Homo sapiens
**ON022570.1**	Brazil	2017	Non-human primate	**KY885001.2**	Brazil	2017	Alouatta guariba clamitans
**ON022706.1**	Brazil	2017	Alouatta sp	**MF170978.1**	Brazil	2017	Homo sapiens
**MH030055.1**	Brazil	2017	Alouatta sp	**MF170979.1**	Brazil	2017	Homo sapiens
**ON022652.1**	Brazil	2017	Non-human primate	**MF170976.1**	Brazil	2017	Homo sapiens
**ON022674.1**	Brazil	2018	Alouatta sp	**ON502402.1**	Brazil	2017	Homo sapiens
**ON022673.1**	Brazil	2018	Alouatta sp	**MF170968.1**	Brazil	2017	Homo sapiens
**ON022347.1**	Brazil	2018	Alouatta sp	**ON022246.1**	Brazil	2018	Homo sapiens
**ON022358.1**	Brazil	2018	Alouatta sp	**ON022245.1**	Brazil	2018	Homo sapiens
**ON022338.1**	Brazil	2018	Alouatta sp	**ON502417.1**	Brazil	2018	Homo sapiens
**ON022329.1**	Brazil	2018	Alouatta sp	**ON022393.1**	Brazil	2017	Callithrix penicillata
**ON022345.1**	Brazil	2018	Alouatta sp	**ON022267.1**	Brazil	2018	Homo sapiens
**MH030067.1**	Brazil	2017	Alouatta sp	**ON022380.1**	Brazil	2017	Alouatta guariba clamitans
**MH030056.1**	Brazil	2017	Alouatta sp	**ON022244.1**	Brazil	2018	Homo sapiens
**MZ604870.1**	Brazil	2017	Homo sapiens	**ON022243.1**	Brazil	2018	Callithrix
**ON022296.1**	Brazil	2018	Alouatta sp	**ON022248.1**	Brazil	2018	Homo sapiens
**MH030084.1**	Brazil	2017	Alouatta sp	**ON022247.1**	Brazil	2018	Homo sapiens
**MH030073.1**	Brazil	2017	Alouatta sp	**ON502418.1**	Brazil	2018	Homo sapiens
**MH030083.1**	Brazil	2017	Alouatta sp	**ON502390.1**	Brazil	2018	Homo sapiens
**MK583157.1**	Brazil	2018	Homo sapiens	**ON022377.1**	Brazil	2018	Callithrix penicillata
**MZ604842.1**	Brazil	2018	Homo sapiens	**ON502400.1**	Brazil	2018	Homo sapiens
**MK583148.1**	Brazil	2018	Homo sapiens	**MK583154.1**	Brazil	2018	Homo sapiens
**ON022361.1**	Brazil	2018	Alouatta sp	**ON502408.1**	Brazil	2018	Homo sapiens
**ON022343.1**	Brazil	2018	Callithrix	**ON502395.1**	Brazil	2018	Homo sapiens
**MH030075.1**	Brazil	2017	Alouatta sp	**ON502397.1**	Brazil	2018	Homo sapiens
**MH030052.1**	Brazil	2017	Alouatta sp	**ON502407.1**	Brazil	2017	Homo sapiens
**MK583153.1**	Brazil	2018	Homo sapiens	**MK333800.1**	Brazil	2017	Alouatta guariba clamitans
**ON022339.1**	Brazil	2018	Alouatta sp	**KY885000.2**	Brazil	2017	Alouatta guariba clamitans

GenBank Acession	Country	Year	Source	GenBank Acession	Country	Year	Source
**ON022344.1**	Brazil	2018	Alouatta sp	**MF423374.2**	Brazil	2017	Haemagogus janthinomys
**ON022336.1**	Brazil	2018	Alouatta sp	**MF370531.1**	Brazil	2017	Homo sapiens
**ON022335.1**	Brazil	2018	Alouatta sp	**MH018107.1**	Brazil	2017	Homo sapiens
**ON022331.1**	Brazil	2018	Alouatta sp	**MF370537.1**	Brazil	2017	Non-human primate
**ON022333.1**	Brazil	2018	Alouatta sp	**MN643084.1**	Brazil	2018	Homo sapiens
**ON022332.1**	Brazil	2018	Alouatta sp	**MN643083.1**	Brazil	2018	Homo sapiens
**ON022334.1**	Brazil	2018	Alouatta sp	**MF370530.1**	Brazil	2017	Haemagogus janthinomys
**ON022351.1**	Brazil	2018	Alouatta sp	**MF538782.2**	Brazil	2017	Homo sapiens
**ON022697.1**	Brazil	2018	Alouatta sp	**ON502415.1**	Brazil	2018	Homo sapiens
**MH193175.1**	Brazil	2018	Alouatta sp	**MK882605.1**	Brazil	2017	Homo sapiens
**MH030085.1**	Brazil	2017	Alouatta sp	**MK882615.1**	Brazil	2017	Homo sapiens
**MK760663.1**	Netherlands	2018	Homo sapiens	**ON022253.1**	Brazil	2018	Homo sapiens
**MK760662.1**	Netherlands	2018	Homo sapiens	**ON022272.1**	Brazil	2018	Homo sapiens
**MK760666.1**	Netherlands	2018	Homo sapiens	**MH484433.1**	Brazil	2017	Non-human primate
**MK760664.1**	Netherlands	2018	Homo sapiens	**MK882616.1**	Brazil	2017	Homo sapiens
**MK760665.1**	Netherlands	2018	Homo sapiens	**ON022256.1**	Brazil	2018	Homo sapiens
**MK760660.1**	Netherlands	2018	Homo sapiens	**ON022249.1**	Brazil	2018	Homo sapiens
**MK760661.1**	Netherlands	2018	Homo sapiens	**MK882599.1**	Brazil	2017	Homo sapiens
**ON022282.1**	Brazil	2018	Homo sapiens	**ON022241.1**	Brazil	2018	Alouatta sp
**MK583158.1**	Brazil	2018	Homo sapiens	**MK882606.1**	Brazil	2017	Homo sapiens
**MK583165.1**	Brazil	2018	Homo sapiens	**MK882608.1**	Brazil	2017	Homo sapiens
**ON022357.1**	Brazil	2017	Alouatta sp	**ON022279.1**	Brazil	2018	Homo sapiens
**MK583149.1**	Brazil	2018	Homo sapiens	**MK882602.1**	Brazil	2017	Domingos Martins
**MH030076.1**	Brazil	2017	Alouatta sp	**ON022261.1**	Brazil	2018	Homo sapiens
**MH030054.1**	Brazil	2017	Alouatta sp	**MH018069.1**	Brazil	2017	Homo sapiens
**MH030069.1**	Brazil	2017	Monkey	**MK882612.1**	Brazil	2017	Homo sapiens
**MK583167.1**	Brazil	2018	Homo sapiens	**MH329655.1**	Brazil	2018	Aedes albopictus
**ON022610.1**	Brazil	2017	Alouatta sp	**MK882609.1**	Brazil	2017	Homo sapiens
**ON022632.1**	Brazil	2017	Alouatta sp	**MH484428.1**	Brazil	2017	Homo sapiens
**ON022315.1**	Brazil	2018	Homo sapiens	**MH484424.1**	Brazil	2017	Homo sapiens
**ON022314.1**	Brazil	2018	Non-human primate	**MH018099.1**	Brazil	2017	Non-human primate
**MH030053.1**	Brazil	2017	Alouatta sp	**MN604280.1**	Brazil	2017	Callithrix
**ON022596.1**	Brazil	2017	Alouatta sp	**MN604287.1**	Brazil	2017	Alouatta sp
**ON022556.1**	Brazil	2019	Homo sapiens	**MH018080.1**	Brazil	2017	Homo sapiens
**ON022557.1**	Brazil	2019	Homo sapiens	**ON502425.1**	Brazil	2017	Homo sapiens
**ON022547.1**	Brazil	2019	Homo sapiens	**MH484425.1**	Brazil	2017	Homo sapiens
**ON022545.1**	Brazil	2019	Homo sapiens	**ON502434.1**	Brazil	2017	Homo sapiens
**ON022560.1**	Brazil	2019	Homo sapiens	**ON502430.1**	Brazil	2017	Homo sapiens
**ON022563.1**	Brazil	2019	Homo sapiens	**ON502427.1**	Brazil	2017	Homo sapiens
**ON022550.1**	Brazil	2019	Homo sapiens	**MH018068.1**	Brazil	2017	Homo sapiens
**ON022676.1**	Brazil	2019	Non-human primate	**MH560359.1**	Brazil	2017	Homo sapiens
**ON022551.1**	Brazil	2019	Homo sapiens	**ON502428.1**	Brazil	2017	Homo sapiens

GenBank Acession	Country	Year	Source	GenBank Acession	Country	Year	Source
**ON022370.1**	Brazil	2019	Non-human primate	**MF370532.1**	Brazil	2017	Homo sapiens
**ON022367.1**	Brazil	2019	Alouatta sp	**MF370534.1**	Brazil	2016	Callithrix penicillata
**ON022368.1**	Brazil	2019	Non-human primate	**ON502437.1**	Brazil	2017	Homo sapiens
**ON022324.1**	Brazil	2018	Alouatta sp	**MF370538.1**	Brazil	2017	Callithrix penicillata
**ON022323.1**	Brazil	2018	Alouatta sp	**MN604281.2**	Brazil	2017	Alouatta sp
**ON022365.1**	Brazil	2018	Alouatta sp	**MN604282.2**	Brazil	2017	Callithrix
**ON022362.1**	Brazil	2017	Alouatta sp	**MN604283.2**	Brazil	2017	Callithrix
**ON022341.1**	Brazil	2018	Alouatta sp	**ON022458.1**	Brazil	2018	Alouatta sp
**ON022363.1**	Brazil	2017	Alouatta sp	**MN604285.1**	Brazil	2017	Callithrix penicillata
**ON022364.1**	Brazil	2017	Alouatta sp	**MN604286.1**	Brazil	2017	Callithrix penicillata
**ON022569.1**	Brazil	2018	Non-human primate	**ON022457.1**	Brazil	2018	Callithrix
**ON022326.1**	Brazil	2018	Alouatta sp	**ON022451.1**	Brazil	2018	Alouatta sp
**ON022328.1**	Brazil	2018	Alouatta sp	**ON022472.1**	Brazil	2018	Homo sapiens
**ON022740.1**	Brazil	2018	Homo sapiens	**ON022464.1**	Brazil	2017	Non-human primate
**ON022327.1**	Brazil	2018	Alouatta sp	**ON022470.1**	Brazil	2018	Homo sapiens
**ON022325.1**	Brazil	2018	Alouatta sp	**ON022447.1**	Brazil	2017	Homo sapiens
**ON022320.1**	Brazil	2018	Alouatta sp	**ON022444.1**	Brazil	2017	Alouatta sp
**ON022602.1**	Brazil	2018	Alouatta sp	**ON022440.1**	Brazil	2018	Alouatta sp
**ON022321.1**	Brazil	2017	Alouatta sp	**MN643092.1**	Brazil	2018	Alouatta guariba
**ON022648.1**	Brazil	2017	Alouatta sp	**ON022459.1**	Brazil	2018	Callithrix
**ON022737.1**	Brazil	2018	Homo sapiens	**ON022434.1**	Brazil	2018	Alouatta sp
**ON022739.1**	Brazil	2018	Homo sapiens	**ON022521.1**	Brazil	2017	Homo sapiens
**ON022741.1**	Brazil	2018	Homo sapiens	**ON022433.1**	Brazil	2018	Alouatta sp
**ON022664.1**	Brazil	2017	Non-human primate	**ON022441.1**	Brazil	2018	Callicebus
**ON022633.1**	Brazil	2017	Alouatta sp	**ON022478.1**	Brazil	2018	Homo sapiens
**ON022715.1**	Brazil	2017	Alouatta sp	**ON022488.1**	Brazil	2018	Homo sapiens
**ON022638.1**	Brazil	2017	Callicebus	**ON022515.1**	Brazil	2018	Homo sapiens
**ON022613.1**	Brazil	2017	Alouatta sp	**ON022395.1**	Brazil	2017	Alouatta guariba clamitans
**ON022712.1**	Brazil	2017	Alouatta sp	**ON022518.1**	Brazil	2017	Non-human primate
**ON022620.1**	Brazil	2017	Alouatta sp	**ON022487.1**	Brazil	2018	Homo sapiens
**ON022711.1**	Brazil	2017	Alouatta sp	**ON022505.1**	Brazil	2018	Homo sapiens
**ON022736.1**	Brazil	2018	Homo sapiens	**MK882613.1**	Brazil	2017	Homo sapiens
**ON022607.1**	Brazil	2017	Alouatta sp	**ON022484.1**	Brazil	2018	Homo sapiens
**ON022713.1**	Brazil	2017	Callithrix	**ON022446.1**	Brazil	2017	Non-human primate
**ON022601.1**	Brazil	2017	Alouatta sp	**ON022436.1**	Brazil	2018	Alouatta sp
**ON022568.1**	Brazil	2017	Alouatta sp	**ON022277.1**	Brazil	2018	Homo sapiens
**ON022663.1**	Brazil	2017	Non-human primate	**MN506278.1**	Brazil	2017	Haemagogus leucocelaenus
**ON022653.1**	Brazil	2017	Non-human primate	**ON022313.1**	Brazil	2018	Alouatta sp
**ON022571.1**	Brazil	2017	Non-human primate	**MN506277.1**	Brazil	2017	Haemagogus leucocelaenus
**ON022667.1**	Brazil	2017	Non-human primate	**MK333802.1**	Brazil	2017	Haemagogus leucocelaenus
**ON022654.1**	Brazil	2017	Non-human primate	**MF170974.1**	Brazil	2017	Homo sapiens
**ON022670.1**	Brazil	2018	Non-human primate	**MN506281.1**	Brazil	2017	Haemagogus janthinomys

GenBank Acession	Country	Year	Source	GenBank Acession	Country	Year	Source
**ON022649.1**	Brazil	2017	Non-human primate	**MN643088.1**	Brazil	2017	Alouatta guariba
**ON022666.1**	Brazil	2017	Non-human primate	**MF423376.2**	Brazil	2017	Alouatta guariba clamitans
**ON022604.1**	Brazil	2017	Alouatta sp	**MF370547.1**	Brazil	2017	Alouatta sp
**ON022624.1**	Brazil	2017	Alouatta sp	**MH018112.1**	Brazil	2017	Alouatta sp
**ON022627.1**	Brazil	2017	Alouatta sp	**MK333805.1**	Brazil	2018	Sabethes chloropterus
**ON022630.1**	Brazil	2017	Alouatta sp	**ON022477.1**	Brazil	2018	Homo sapiens
**ON022707.1**	Brazil	2017	Alouatta sp	**ON022490.1**	Brazil	2018	Homo sapiens
**ON022655.1**	Brazil	2017	Alouatta sp	**ON022496.1**	Brazil	2017	Homo sapiens
**ON022622.1**	Brazil	2017	Alouatta sp	**ON022453.1**	Brazil	2018	Callithrix
**OP508590.1**	Brazil	2019	Homo sapiens	**ON022287.1**	Brazil	2018	Alouatta sp
**ON022647.1**	Brazil	2017	Alouatta sp	**MN643086.1**	Brazil	2018	Homo sapiens
**ON022605.1**	Brazil	2017	Alouatta sp	**MH378284.1**	Brazil	2017	Homo sapiens
**MH030058.1**	Brazil	2017	Alouatta sp	**ON022285.1**	Brazil	2018	Alouatta sp
**MH030068.1**	Brazil	2017	Alouatta sp	**MN506270.1**	Brazil	2017	Haemagogus leucocelaenus
**MH030066.1**	Brazil	2017	Alouatta sp	**MN506269.1**	Brazil	2017	Haemagogus leucocelaenus
**ON022390.1**	Brazil	2017	Alouatta caraya	**MN506274.1**	Brazil	2017	Aedes taeniorhynchus
**ON022381.1**	Brazil	2017	Alouatta caraya	**MN506272.1**	Brazil	2017	Aedes scapularis
**ON022646.1**	Brazil	2017	Alouatta sp	**MK089775.1**	Brazil	2017	Ochlerotatus taeniorhynchus
**ON022621.1**	Brazil	2017	Non-human primate	**MN506276.1**	Brazil	2017	Haemagogus leucocelaenus
**MH030087.1**	Brazil	2017	Homo sapiens	**MF538786.2**	Brazil	2017	marmoset
**ON022685.1**	Brazil	2017	Alouatta sp	**MN643081.1**	Brazil	2018	Homo sapiens
**MZ604865.1**	Brazil	2018	Homo sapiens	**ON022480.1**	Brazil	2018	Homo sapiens
**MZ604856.1**	Brazil	2018	Homo sapiens	**MN643082.1**	Brazil	2018	Homo sapiens
**MK583161.1**	Brazil	2018	Homo sapiens	**MK882603.1**	Brazil	2018	Homo sapiens
**MK583162.1**	Brazil	2018	Homo sapiens	**MK882600.1**	Brazil	2018	Homo sapiens
**MK583175.1**	Brazil	2018	Homo sapiens	**MK533792.1**	Brazil	2019	Alouatta guariba clamitans
**ON022744.1**	Brazil	2018	Homo sapiens	**MN506275.1**	Brazil	2017	Haemagogus leucocelaenus
**MK583182.1**	Brazil	2018	Homo sapiens	**MF170981.1**	Brazil	2017	Homo sapiens
**MH193174.1**	Brazil	2018	Homo sapiens	**MF170970.1**	Brazil	2017	Homo sapiens
**MH030078.1**	Brazil	2017	Alouatta sp	**MF170969.1**	Brazil	2017	Homo sapiens
**MZ604876.1**	Brazil	2018	Homo sapiens	**MN506280.1**	Brazil	2017	Haemagogus leucocelaenus
**MK583176.1**	Brazil	2018	Homo sapiens	**MN506279.1**	Brazil	2017	Haemagogus leucocelaenus
**ON022586.1**	Brazil	2018	Alouatta sp	**MN506268.1**	Brazil	2017	Haemagogus leucocelaenus
**ON022294.1**	Brazil	2018	Haemagogus leucocelaenus	**MN506271.1**	Brazil	2017	Haemagogus leucocelaenus
**ON022291.1**	Brazil	2018	Haemagogus leucocelaenus	**MN506267.1**	Brazil	2017	Haemagogus leucocelaenus
**ON022293.1**	Brazil	2018	Haemagogus leucocelaenus	**MF170980.1**	Brazil	2017	Homo sapiens
**ON022304.1**	Brazil	2018	Haemagogus leucocelaenus	**MK333801.1**	Brazil	2017	Haemagogus janthinomys
**MH030072.1**	Brazil	2017	Alouatta sp	**MF423375.2**	Brazil	2017	Alouatta guariba clamitans
**ON022588.1**	Brazil	2018	Alouatta sp	**MK583168.1**	Brazil	2018	Homo sapiens
**MK583156.1**	Brazil	2018	Homo sapiens	**MN506282.1**	Brazil	2017	Haemagogus janthinomys
**MK583181.1**	Brazil	2018	Homo sapiens	**MF170973.1**	Brazil	2017	Homo sapiens
**ON022284.1**	Brazil	2018	Callithrix	**MN643091.1**	Brazil	2018	Alouatta guariba

GenBank Acession	Country	Year	Source	GenBank Acession	Country	Year	Source
**MH030088.1**	Brazil	2017	Homo sapiens	**MN506283.1**	Brazil	2017	Haemagogus janthinomys
**MK583179.1**	Brazil	2018	Homo sapiens	**MN506273.1**	Brazil	2017	Haemagogus leucocelaenus
**MK583152.1**	Brazil	2017	Homo sapiens	**MN643090.1**	Brazil	2018	Alouatta guariba
**MK583178.1**	Brazil	2018	Homo sapiens	**MK882611.1**	Brazil	2017	Homo sapiens
**ON022298.1**	Brazil	2018	Homo sapiens	**MK882607.1**	Brazil	2017	Homo sapiens
**MK583164.1**	Brazil	2018	Homo sapiens	**MF170972.1**	Brazil	2017	Homo sapiens
**MZ604868.1**	Brazil	2018	Homo sapiens	**MK882621.1**	Brazil	2018	Homo sapiens
**ON022592.1**	Brazil	2018	Alouatta sp	**MF434851.2**	Brazil	2017	Homo sapiens
**ON022577.1**	Brazil	2018	Alouatta sp	**MN643089.1**	Brazil	2017	Alouatta guariba
**ON022675.1**	Brazil	2018	Alouatta sp	**ON022435.1**	Brazil	2017	Alouatta sp
**ON022626.1**	Brazil	2017	Alouatta sp	**ON022269.1**	Brazil	2017	Alouatta sp
**ON022340.1**	Brazil	2018	Alouatta sp	**MK882617.1**	Brazil	2017	Homo sapiens
**ON022348.1**	Brazil	2018	Alouatta sp	**MN643087.1**	Brazil	2018	Homo sapiens
**ON022318.1**	Brazil	2018	Homo sapiens	**MN506266.1**	Brazil	2018	Haemagogus janthinomys
**ON022346.1**	Brazil	2018	Alouatta sp	**MN643085.1**	Brazil	2018	Homo sapiens
**ON022350.1**	Brazil	2018	Alouatta sp	**MF538783.2**	Brazil	2017	Homo sapiens
**MH030089.1**	Brazil	2017	Homo sapiens	**MF370533.1**	Brazil	2017	Homo sapiens
**ON022283.1**	Brazil	2018	Callicebus	**ON502432.1**	Brazil	2017	Homo sapiens
**ON022668.1**	Brazil	2018	Non-human primate	**ON502404.1**	Brazil	2017	Homo sapiens
**ON022292.1**	Brazil	2018	Non-human primate	**ON502426.1**	Brazil	2017	Homo sapiens
**ON022594.1**	Brazil	2018	Alouatta sp	**MH018091.1**	Brazil	2017	Homo sapiens
**ON022637.1**	Brazil	2017	Alouatta sp	**MH018078.1**	Brazil	2017	Homo sapiens
**MK583160.1**	Brazil	2018	Homo sapiens	**ON502429.1**	Brazil	2017	Homo sapiens
**ON022575.1**	Brazil	2017	Non-human primate	**MH018094.1**	Brazil	2017	Homo sapiens
**MK583172.1**	Brazil	2018	Homo sapiens	**MH018110.1**	Brazil	2017	Callithrix sp.
**ON022671.1**	Brazil	2018	Non-human primate	**MH018111.1**	Brazil	2017	Homo sapiens
**ON022565.1**	Brazil	2018	Non-human primate	**MH018113.1**	Brazil	2017	Homo sapiens
**ON022657.1**	Brazil	2018	Alouatta sp	**MH018109.1**	Brazil	2017	Alouatta sp
**ON022629.1**	Brazil	2017	Alouatta sp	**MH018106.1**	Brazil	2017	Alouatta sp
**ON022572.1**	Brazil	2018	Alouatta sp	**MH018104.1**	Brazil	2017	Homo sapiens
**ON022650.1**	Brazil	2017	Alouatta sp	**MH018102.1**	Brazil	2017	Non-human primate
**ON022597.1**	Brazil	2018	Non-human primate	**MH018103.1**	Brazil	2017	Homo sapiens
**ON022635.1**	Brazil	2017	Non-human primate	**MH018105.1**	Brazil	2017	Homo sapiens
**ON022554.1**	Brazil	2018	Homo sapiens	**MK249065.1**	Brazil	2017	Homo sapiens
**ON022672.1**	Brazil	2018	Non-human primate	**MH018096.1**	Brazil	2017	Alouatta sp
**ON022322.1**	Brazil	2018	Non-human primate	**MH018095.1**	Brazil	2017	Callithrix sp
**ON022705.1**	Brazil	2017	Alouatta sp	**MH018079.1**	Brazil	2017	Homo sapiens
**ON022682.1**	Brazil	2017	Non-human primate	**ON502433.1**	Brazil	2017	Homo sapiens
**ON022679.1**	Brazil	2017	Non-human primate	**ON502423.1**	Brazil	2017	Homo sapiens
**ON022680.1**	Brazil	2017	Non-human primate	**ON502436.1**	Brazil	2017	Homo sapiens
**ON022414.1**	Brazil	2017	Alouatta caraya	**ON502431.1**	Brazil	2017	Homo sapiens
**MH018064.1**	Brazil	2017	Alouatta sp	**ON502406.1**	Brazil	2017	Homo sapiens

GenBank Acession	Country	Year	Source	GenBank Acession	Country	Year	Source
**ON022540.1**	Brazil	2018	Homo sapiens	**MF465805.1**	Brazil	2017	Homo sapiens
**ON022541.1**	Brazil	2018	Homo sapiens	**MH484429.1**	Brazil	2017	Callithrix sp
**ON022429.1**	Brazil	2017	Alouatta caraya	**MF370535.1**	Brazil	2016	Alouatta sp
**ON022400.1**	Brazil	2017	Callithrix penicillata	**MF370536.1**	Brazil	2016	Alouatta sp
**ON022600.1**	Brazil	2016	Alouatta sp	**MF370549.1**	Brazil	2015	Non-human primate
**ON022669.1**	Brazil	2018	Callicebus	**MK882614.1**	Brazil	2017	Homo sapiens
**ON022599.1**	Brazil	2018	Cebus	**MH018071.1**	Brazil	2017	Homo sapiens
**ON022689.1**	Brazil	2017	Non-human primate	**ON502439.1**	Brazil	2018	Homo sapiens
**ON022688.1**	Brazil	2016	Alouatta sp	**ON502405.1**	Brazil	2018	Homo sapiens
**MT497521.1**	Brazil	2016	Alouatta sp	**ON022372.1**	Brazil	2017	Alouatta guariba clamitans
**ON022677.1**	Brazil	2017	Sapajus	**ON022251.1**	Brazil	2018	Cebidae
**ON022698.1**	Brazil	2016	Alouatta sp	**ON502440.1**	Brazil	2018	Homo sapiens
**ON022681.1**	Brazil	2017	Callicebus	**ON502411.1**	Brazil	2018	Homo sapiens
**ON022408.1**	Brazil	2017	Alouatta caraya	**ON022255.1**	Brazil	2018	Homo sapiens
**ON022407.1**	Brazil	2017	Alouatta guariba clamitans	**ON022250.1**	Brazil	2018	Homo sapiens
**ON022581.1**	Brazil	2017	Alouatta sp	**ON502420.1**	Brazil	2018	Homo sapiens
**ON022686.1**	Brazil	2016	Alouatta sp	**ON022254.1**	Brazil	2018	Callithrix penicillata
**MT497522.1**	Brazil	2016	Alouatta sp	**ON022252.1**	Brazil	2018	Cebidae
**ON022579.1**	Brazil	2017	Alouatta sp	**ON502421.1**	Brazil	2018	Homo sapiens
**ON022421.1**	Brazil	2017	Alouatta caraya	**ON502441.1**	Brazil	2018	Homo sapiens
**ON022426.1**	Brazil	2017	Cebidae	**ON502401.1**	Brazil	2018	Homo sapiens
**MT497525.1**	Brazil	2017	Alouatta sp	**ON022371.1**	Brazil	2017	Alouatta caraya
**ON022703.1**	Brazil	2016	Alouatta sp	**ON022410.1**	Brazil	2017	Callithrix penicillata
**ON022591.1**	Brazil	2018	Alouatta sp	**ON022403.1**	Brazil	2017	Alouatta guariba clamitans
**ON022587.1**	Brazil	2018	Alouatta sp	**ON022402.1**	Brazil	2017	Non-human primate
**ON022289.1**	Brazil	2018	Hg janthinomys capricornii	**ON022419.1**	Brazil	2017	Non-human primate
**ON022286.1**	Brazil	2018	Hg janthinomys capricornii	**ON022391.1**	Brazil	2017	Alouatta guariba clamitans
**ON022305.1**	Brazil	2018	Hg janthinomys capricornii	**ON022376.1**	Brazil	2018	Alouatta sp
**MH030086.1**	Brazil	2017	Alouatta sp	**ON022412.1**	Brazil	2017	Alouatta caraya
**ON022593.1**	Brazil	2018	Non-human primate	**ON022394.1**	Brazil	2018	Callithrix penicillata
**ON022312.1**	Brazil	2018	Callithrix	**ON022404.1**	Brazil	2017	Alouatta caraya
**ON022584.1**	Brazil	2018	Alouatta sp	**ON022396.1**	Brazil	2017	Callithrix penicillata
**MK583177.1**	Brazil	2018	Homo sapiens	**ON022418.1**	Brazil	2017	Sapajus apella
**MH030082.1**	Brazil	2017	Alouatta sp	**ON022401.1**	Brazil	2017	Non-human primate
**MH030081.1**	Brazil	2017	Alouatta sp	**ON022389.1**	Brazil	2017	Cebidae
**MH030079.1**	Brazil	2017	Alouatta sp	**MH484431.1**	Brazil	2017	Non-human primate
**ON022316.1**	Brazil	2018	Homo sapiens	**MH666058.1**	Brazil	2016	Sapajus sp
**ON022619.1**	Brazil	2017	Alouatta sp	**MH666056.1**	Brazil	2016	Alouatta sp
**ON022310.1**	Brazil	2018	Homo sapiens	**ON022288.1**	Brazil	2016	Non-human primate
**ON022290.1**	Brazil	2018	Haemagogus leucocelaenus	**ON022700.1**	Brazil	2016	Alouatta sp
**ON022580.1**	Brazil	2017	Alouatta sp	**MT497523.1**	Brazil	2016	Alouatta sp
**ON022615.1**	Brazil	2017	Alouatta sp	**MT497524.1**	Brazil	2016	Alouatta sp

GenBank Acession	Country	Year	Source	GenBank Acession	Country	Year	Source
**ON022704.1**	Brazil	2017	Non-human primate	**ON022311.1**	Brazil	2016	Alouatta sp
**ON022614.1**	Brazil	2017	Alouatta sp	**MT497520.1**	Brazil	2016	Sapajus sp
**ON022386.1**	Brazil	2017	Alouatta caraya	**OP508588.1**	Brazil	2022	Non-human primate
**ON022693.1**	Brazil	2017	Non-human primate	**OP508585.1**	Brazil	2022	Non-human primate
**ON022611.1**	Brazil	2017	Non-human primate	**OP508582.1**	Brazil	2022	Non-human primate
**ON022573.1**	Brazil	2017	Alouatta sp	**OP508583.1**	Brazil	2022	Non-human primate
**ON022609.1**	Brazil	2017	Alouatta sp	**OP508586.1**	Brazil	2022	Non-human primate
**ON022566.1**	Brazil	2017	Alouatta sp	**OP508581.1**	Brazil	2022	Non-human primate
**ON022618.1**	Brazil	2017	Non-human primate	**OP508589.1**	Brazil	2022	Non-human primate
**ON022692.1**	Brazil	2017	Alouatta sp	**OP508580.1**	Brazil	2022	Non-human primate
**ON022690.1**	Brazil	2017	Alouatta sp	**OP508578.1**	Brazil	2022	Non-human primate
**ON022684.1**	Brazil	2017	Sapajus	**OP508584.1**	Brazil	2022	Non-human primate
**ON022691.1**	Brazil	2017	Non-human primate	**OP508577.1**	Brazil	2022	Non-human primate
**ON022616.1**	Brazil	2017	Alouatta sp	**OP508579.1**	Brazil	2022	Non-human primate
**ON022639.1**	Brazil	2017	Callithrix	**OL519589.1**	Brazil	2021	Alouatta caraya
**ON022589.1**	Brazil	2018	Alouatta sp	**OL519588.1**	Brazil	2021	Alouatta caraya
**ON022694.1**	Brazil	2018	Alouatta sp	**OL519587.1**	Brazil	2021	Alouatta caraya
**ON022623.1**	Brazil	2017	Alouatta sp	**OQ572696.1**	Brazil	2021	Sabethes chloropterus
**ON022378.1**	Brazil	2017	Cebidae	**OQ572695.1**	Brazil	2021	Sabethes chloropterus
**ON022661.1**	Brazil	2018	Non-human primate	**OP508576.1**	Brazil	2021	Non-human primate
**ON022710.1**	Brazil	2017	Alouatta sp	**OP508574.1**	Brazil	2020	Alouatta caraya
**ON022578.1**	Brazil	2017	Alouatta sp	**OP508571.1**	Brazil	2020	Callithrix penicillata
**ON022617.1**	Brazil	2017	Alouatta sp	**OP508572.1**	Brazil	2020	Alouatta caraya
**ON022590.1**	Brazil	2018	Alouatta sp	**OP508570.1**	Brazil	2020	Callithrix penicillata
**ON022687.1**	Brazil	2017	Alouatta sp	**OP508575.1**	Brazil	2020	Alouatta caraya
**ON022582.1**	Brazil	2017	Cebus	**OP508573.1**	Brazil	2020	Alouatta caraya
**MH030077.1**	Brazil	2017	Alouatta sp	**OP508715.1**	Brazil	2017	Non-human primate
**MH030090.1**	Brazil	2017	Homo sapiens	**MF370546.1**	Brazil	2017	Alouatta caraya
**ON022317.1**	Brazil	2018	Homo sapiens	**MF370544.1**	Brazil	2017	Aotus ozzarae infulatus
**ON022612.1**	Brazil	2017	Non-human primate	**MF370540.1**	Brazil	2017	Alouatta seniculus
**ON022708.1**	Brazil	2017	Alouatta sp	**MF370541.1**	Brazil	2017	Alouatta sp.
**ON022738.1**	Brazil	2018	Homo sapiens	**O-022716.1**	Brazil	2004	Homo sapiens
**ON022576.1**	Brazil	2017	Alouatta sp	**MW158352.1**	Venezuela	2005	Alouatta seniculus
**ON022585.1**	Brazil	2018	Cebus	**MW158351.1**	Venezuela	2005	Alouatta seniculus
**MH018066.1**	Brazil	2017	Alouatta sp	**MW158355.1**	Venezuela	2006	Alouatta seniculus
**MH018067.1**	Brazil	2017	Cebidae sp	**KM388818.1**	Venezuela	2006	Alouatta seniculus
**ON022374.1**	Brazil	2017	Alouatta sp	**MW158356.1**	Venezuela	2007	Alouatta seniculus
**MW034590.1**	Brazil	2016	Alouatta caraya	**KM388815.1**	Venezuela	2007	Alouatta seniculus
**ON022303.1**	Brazil	2017	Non-human primate	**KM388814.1**	Venezuela	2005	Homo sapiens
**ON022699.1**	Brazil	2017	Alouatta sp	**MW158353.1**	Venezuela	2005	Homo sapiens
**ON022696.1**	Brazil	2019	Alouatta sp	**MW158354.1**	Venezuela	2005	Homo sapiens
**ON022651.1**	Brazil	2017	Alouatta sp	**MW158357.1**	Venezuela	2010	Alouatta seniculus

GenBank Acession	Country	Year	Source	GenBank Acession	Country	Year	Source
**ON022542.1**	Brazil	2018	Homo sapiens	**KM388816.1**	Venezuela	2010	Alouatta seniculus
**ON022656.1**	Brazil	2018	Alouatta sp	**MW158350.1**	Venezuela	2004	Alouatta seniculus
**ON022640.1**	Brazil	2017	Alouatta sp	**KM388817.1**	Venezuela	2004	Alouatta seniculus
**ON022643.1**	Brazil	2017	Alouatta sp	**MK333803.1**	Brazil	2017	Alouatta caraya
**ON022683.1**	Brazil	2017	Non-human primate	**MF370543.1**	Brazil	2017	Alouatta sp.
**ON022608.1**	Brazil	2017	Alouatta sp	**KY861728.1**	Brazil	2008	Alouatta sp.
**ON022662.1**	Brazil	2017	Alouatta sp	**JF912190.1**	Brazil	2002	Homo sapiens
**ON022641.1**	Brazil	2017	Alouatta sp	**MF370542.1**	Brazil	2017	Alouatta guariba
**ON022606.1**	Brazil	2017	Non-human primate	**MF370539.1**	Brazil	2017	Sapajus libidinosus
**ON022603.1**	Brazil	2018	Alouatta sp	**ON022717.1**	Brazil	2000	Homo sapiens
**ON022366.1**	Brazil	2018	Alouatta sp	**ON022720.1**	Brazil	2000	Homo sapiens
**ON022665.1**	Brazil	2017	Non-human primate	**JF912188.1**	Brazil	2000	Homo sapiens
**ON022695.1**	Brazil	2018	Callithrix	**MH018101.1**	Brazil	2003	Homo sapiens
**ON022746.1**	Brazil	2018	Homo sapiens	**MH018100.1**	Brazil	2003	Homo sapiens
**MH030070.1**	Brazil	2017	Alouatta sp	**MG969501.1**	Brazil	2001	Homo sapiens
**MH030057.1**	Brazil	2017	Alouatta sp	**JF912187.1**	Brazil	2000	Homo sapiens
**MK583169.1**	Brazil	2018	Homo sapiens	**MW158342.1**	Brazil	2000	Homo sapiens
**MZ604873.1**	Brazil	2018	Homo sapiens	**ON022718.1**	Brazil	2000	Homo sapiens
**MK583180.1**	Brazil	2018	Homo sapiens	**JF912189.1**	Brazil	2001	Alouatta sp.
**MK583174.1**	Brazil	2018	Homo sapiens	**MF370545.1**	Brazil	2017	Homo sapiens
**MK583159.1**	Brazil	2018	Homo sapiens	**MF347613.1**	Suriname	2017	Homo sapiens
**MK583171.1**	Brazil	2018	Homo sapiens	**MW158367.1**	Trinidad and Tobago	2009	Coquillettidia venezuelensis
**MK583170.1**	Brazil	2018	Homo sapiens	**MW158365.1**	Trinidad and Tobago	2008	Mansonia titillans
**ON022299.1**	Brazil	2018	Alouatta sp	**HM582851.1**	Trinidad and Tobago	2009	Alouatta seniculus
**MH030071.1**	Brazil	2017	Alouatta sp	**MW158366.1**	Trinidad and Tobago	2009	Culex spissipes
**MK583150.1**	Brazil	2018	Homo sapiens	**MW158364.1**	Trinidad and Tobago	2009	Coquillettidia venezuelensis
**ON022297.1**	Brazil	2018	Sapajus	**MW158349.1**	Venezuela	2004	Alouatta seniculus
**MH030074.1**	Brazil	2017	Alouatta sp	**MW158348.1**	Trinidad and Tobago	1995	Haemagogus sp.
**MK583173.1**	Brazil	2018	Homo sapiens	**MW158346.1**	Trinidad and Tobago	1989	Sabethes chloropterus
**ON022349.1**	Brazil	2018	Cebus	**MW158345.1**	Trinidad and Tobago	1988	Haemagogus janthinomys
**ON022625.1**	Brazil	2017	Alouatta sp	**MW158347.1**	Trinidad and Tobago	1989	Alouatta sp.
**MZ604861.1**	Brazil	2018	Homo sapiens	**ON022722.1**	Brazil	1993	Homo sapiens
**MH030080.1**	Brazil	2017	Alouatta sp	**JF912185.1**	Brazil	1992	Sabethes sp.
**ON022742.1**	Brazil	2018	Homo sapiens	**JF912182.1**	Brazil	1984	Homo sapiens
**MZ604866.1**	Brazil	2018	Homo sapiens	**MW158344.1**	Brazil	1985	Haemagogus janthinomys
**MZ604840.1**	Brazil	2018	Homo sapiens	**JF912180.1**	Brazil	1981	Homo sapiens
**ON022574.1**	Brazil	2017	Alouatta sp	**MW158343.1**	Brazil	1973	Haemagogus sp.
**ON022701.1**	Brazil	2016	Alouatta sp	**MW158341.1**	Brazil	1991	Alouatta sp.
**MN117917.1**	Brazil	2017	Ochlerotatus scapularis	**JF912186.1**	Brazil	1994	Homo sapiens
**ON022702.1**	Brazil	2016	Sapajus	**JF912184.1**	Brazil	1987	Homo sapiens
**ON022301.1**	Brazil	2016	mosquito	**JF912179.1**	Brazil	1980	Haemagogus sp.
**ON022302.1**	Brazil	2016	mosquito	**JF912183.1**	Brazil	1984	Homo sapiens

GenBank Acession	Country	Year	Source	GenBank Acession	Country	Year	Source
**MH666057.1**	Brazil	2017	Alouatta sp	**MW158340.1**	Brazil	1968	Saguinus midas
**MH666060.1**	Brazil	2017	Haemagogus janthinomys	**MW158338.1**	Brazil	1955	Sentinel monkey
**MH666059.1**	Brazil	2017	Haemagogus leucocelaenus	**MW158339.1**	Brazil	1960	Cebus sp.
**ON022300.1**	Brazil	2017	mosquito	**MW158361.1**	Peru	1995	Homo sapiens
**MH018082.1**	Brazil	2017	Callithrix sp	**MW158359.1**	Peru	1998	Homo sapiens
**MH018065.1**	Brazil	2017	Cebidae sp	**MF004382.1**	Bolivia	1999	Homo sapiens
**JF912181.1**	Brazil	1983	Homo sapiens	**KF907504.1**	Bolivia	1999	Homo sapiens

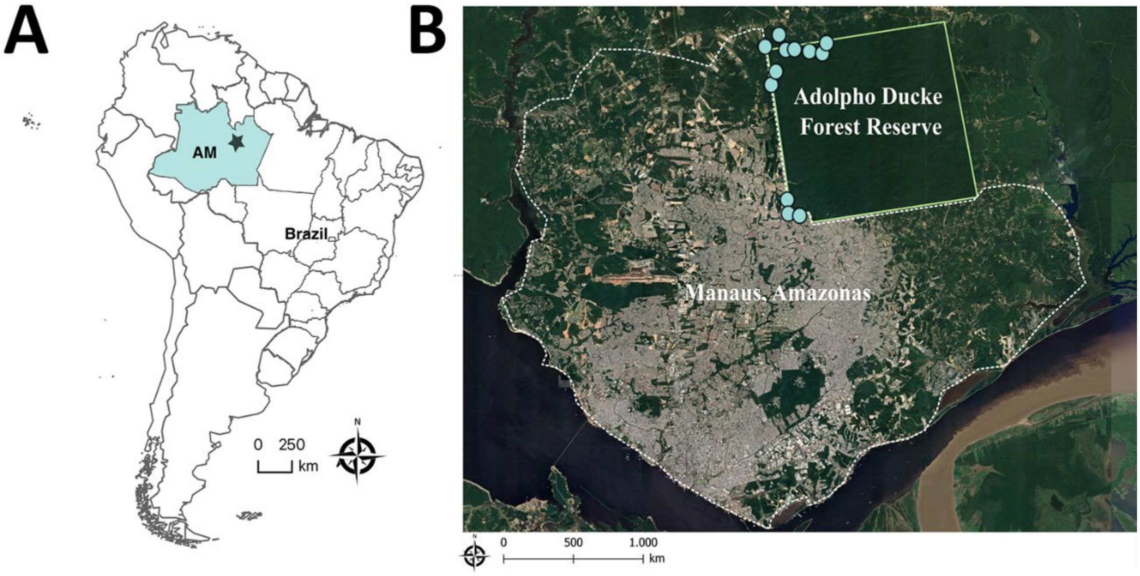

Appendix Figure. Study area related to investigation of yellow fever virus in mosquitoes from rainforest bordering Manaus, Brazil, 2022. A) Location of Manaus in Brazil. B) The Adolpho Ducke Forest Reserve bordering the city of Manaus, with location of sampling sites (blue dots).
